# Editorial for the Special Issue “Cognitive Impairment and Neuropsychiatric Dysfunctions in Multiple Sclerosis (Volume II)”

**DOI:** 10.3390/neurosci7040087

**Published:** 2026-08-03

**Authors:** Ugo Nocentini

**Affiliations:** 1 LIFE Institute for Research and Health Care Santa Lucia IRCCS, Via Ardeatina 306/354, 00179 Roma, Italy; u.nocentini@hsantalucia.it; 2 Department of Clinical Sciences and Translational Medicine, University of Rome “Tor Vergata”, 00133 Rome, Italy

## 1. Introduction

Multiple sclerosis (MS) is the second-leading cause of disability in young adults. It occurs during that stage of life when people often start a family, begin a career, and actively participate in their community and society: a crucial phase in a person’s life.

MS is a proteomic disease that can cause impairment of every function of the central nervous system: deficits in somatic sensation, voluntary and reflex motility, balance, vision, bladder and bowel function, cognitive and affective functions. It is considered an autoimmune disease characterized by inflammatory, demyelinating, and degenerative processes [1,2].

The prognosis of MS has changed over the past 30 years thanks to the availability of increasingly effective disease-modifying drugs [1,2].

Despite this, a considerable percentage of people with MS develop multiple significant disabilities.

The disorders caused by MS can be divided into a group of evident manifestations (e.g., motor symptoms) and another of invisible symptoms [3,4]. Among the latter, cognitive and psychiatric symptoms are certainly prominent: cognitive impairment, with quantitatively and qualitatively different profiles, affects 43 to 70% of people with MS [5]; among psychiatric disturbances, depression and anxiety disorders stand out, but bipolar disorder, personality disorders, and psychosis also appear more common than in the general population [6].

The *NeuroSci* volume introduced by this editorial is, in fact, dedicated to the assessment, consequences and effectiveness of certain treatments of cognitive impairment in MS. Over the past 20 years, knowledge about cognitive and affective disorders has increased significantly; however, several aspects remain unclear [7,8]. On the one hand, there are no shared opinions on assessment methods for these disorders; above all, the question of optimizing resources, both time and personnel, to be used for these assessments remains unresolved.

Another important issue, relating to cognitive disorders, concerns the effectiveness of the treatments offered and what is important in terms of patient outcomes. Even with regard to therapies for cognitive disorders, there is a problem regarding the possibility of providing them to people who have difficulty leaving their homes: this requires experimenting with alternative treatment strategies.

The articles in this volume examine aspects more or less directly related to the topic of cognitive disorders: the three central papers are that by Balconi et al. [9] on new approaches to assessing cognitive function in people with MS, that by Manocchio et al. [10], a narrative review of the impact of cognitive re-education on quality of life and psychological well-being, and that by Herrera-Rojas et al. [11] on the effects of tele-rehabilitation platforms on the quality of life of people with MS.

Balconi et al. [9] examine the landscape of cognitive assessment tools for people with MS in light of recent advances in the field. While many assessment tools are available, the search for new approaches is driven by the constraints of time and resources for conducting assessments. Therefore, the paper devotes due attention to tools that can be administered both in pen-and-paper and computer-based formats. This comprehensive analysis provides, on the one hand, additional data supporting the use of the Brief International Cognitive Assessment for MS (BICAMS) [12], for which versions validated in numerous linguistic and cultural contexts are available. On the other hand, the reader is offered the most recent computerized versions of single tests, primarily inspired by the Symbol Digit Modalities Test, as well as a comprehensive series of new tests and short batteries whose validity and utility will be established over time. Thus, the work by Balconi et al. [9] represents a useful and up-to-date guide to the vast landscape of cognitive assessment methods in MS.

The review by Manocchio et al. [10] focuses on the potential benefits of cognitive re-education on the quality of life and psychological well-being of people with MS. Various cognitive re-education approaches have been shown to be effective in people with MS based on improvements in neuropsychological test scores. These results are certainly significant, but the benefits observed in terms of improved test scores are not easily translated into tangible changes in performance in daily living activities. Identifying parameters that directly measure the benefit experienced by the person undergoing re-education allows a step toward the acceptance of a given therapeutic approach as effective and validated. The authors conclude that the most effective re-education approach is the personalized and multidisciplinary one. This is not surprising given the variability of the dysfunctional framework that affects people with MS. These results sustain the opinion that re-education activities have to be entrusted to expert therapists who maintain a direct relationship with the patient. Nonetheless, integrative programs using virtual reality or music therapy appear to provide benefits for both cognitive function and psychosocial well-being.

The review by Herrera-Rojas et al. [11] seems to support a different opinion. This systematic review has evaluated the effects of randomized clinical trials conducted using tele-rehabilitation platforms on the quality of life of MS patients. The interventions implemented in the trials considered in the review are heterogeneous, ranging from video-monitored home exercise programs to activity-monitored programs for increasing manual dexterity, symptom control, and increased physical activity, to the use of virtual reality, and more.

Tele-rehabilitation was found to be more effective than no intervention, equally effective as in-person interventions in people with MS with an EDSS of 6 or less, and superior to in-person interventions in patients with an EDSS between 5.5 and 7.5. These data, however, somewhat support the fact that patient characteristics influence rehabilitation outcomes.

The two reviews of Manocchio et al. [10] and Herrera-Rojas et al. [11] also underline that, in the field of rehabilitation for people with MS, significant research is needed, using very stringent criteria, to identify the effectiveness of various approaches.

The other papers published in the Special Issue are all worthy of attention, as they address relevant aspects of the disease: from the assessment of swallowing function to the impact of MS on work productivity, from nutritional aspects and their impact on quality of life and disability to the connection between oxidative stress, mitochondrial dysfunction, iron metabolism, and microglial function.

Special mention should be made of the systematic review and meta-analysis whose results are described in the paper by Yazdan Panah et al. [13]. The relevance of the MRI finding of Slowly Expanding Lesions (SELs) has increased in recent years, particularly due to its connection with smoldering disease [14], currently considered a condition underlying the accumulation of disability that is not explained by the extent of focal lesions. The data obtained from the review by Panah et al. demonstrate that MRIs of more than half of MS patients show SELs, confirming that this parameter deserves close attention from clinicians in the challenging task of assessing the disease’s course.

## 2. Concluding Remarks

After Charcot’s initial description of multiple sclerosis, which identified the presence of cognitive and affective disorders, these disorders were no longer studied for many years [15]. However, since cognitive and affective disorders have been reconsidered, more and more researchers have devoted attention to them, as these disorders have very serious consequences for MS patients, and there is still considerable work to be done regarding many of their aspects [7]. Some of the papers in this volume contribute to this end, especially by reviewing the literature on certain relevant issues. In this specific field, as in MS in general, there remains ample room for increasing knowledge and finding solutions. Regarding cognitive disorders, the aspects that will receive the most attention are the following: assessment methods and the relationship between cognitive disorders and structural and functional damage caused by MS, as measured by instrumental methods, especially MRI techniques; and pharmacological and re-educational treatments for cognitive disorders. In terms of affective disorders, the most relevant aspect for future studies is to understand whether the various kinds, depression first and foremost, present with different characteristics and etiologies than those found in people without MS.

## Data Availability

No new data were created or analyzed in this study. Data sharing is not applicable to this article.
